# To the Medical Profession of Georgia

**Published:** 1874-10

**Authors:** 


*To the Medical Profession of Georgia.*

The Georgia Medical Society, believing that the law giving grocery-men and boarding-house keepers the right to garnishee wages and salaries of employees to be a good one, and of great benefit to the employee, enabling the former to do a credit-business, and the latter to procure provisions for his family. Believing further, that in case of sickness it is just as necessery that he shonld have prompt medical attendance, which he cannot get unless the physician has some guarantee to pay for his services, the Society has determined to appeal to the Medical Profession of the State, urging them to use their influence with their representatives to the legislature to procure the passage of a law extending to physicians the same right of garnishment that grocery-men and boarding-house keepers now have.

The law referred to is to be found in the Georgia laws for 1872, No. 44-0, No. 91.

AN ACT TO AMEND THE GARNISHMENT LAWS OF THIS STATE.

17. SECTION 1. Be it enacted by the general assembly of the State of Georgia, and it is hereby enacted by the authority of the same.

"That, from and after the passage of this act, the wages of no person in the employment of another shall be exempt from the process of garnishment, when the consideration of the debt is provisions for the use of the employee or his family, or when the consideration of said debt is for board of himself and family.

THEO. STARBUCK, M.D.,
W. H. ELLIOTT, M.D.,
J. C. LEHARDY, M.D.
*Committee.*

We take pleasure in laying the above circular before the profession of Georgia, and trust the fraternity will *instruct* their friends of the general assembly to lend a helping hand in a matter so important to the profession of the State. W. T. G.

